# Comparison and extension of three methods for automated registration of multimodal plant images

**DOI:** 10.1186/s13007-019-0426-8

**Published:** 2019-04-29

**Authors:** Michael Henke, Astrid Junker, Kerstin Neumann, Thomas Altmann, Evgeny Gladilin

**Affiliations:** Leibniz Institute of Plant Genetics and Crop Plant Research (IPK), OT Gatersleben, Corrensstrasse 3, 06466 Seeland, Germany

**Keywords:** Multimodal image registration, Feature-point matching, Phase correlation, Mutual information, Scale space, High-throughput plant phenotyping

## Abstract

With the introduction of high-throughput multisensory imaging platforms, the automatization of multimodal image analysis has become the focus of quantitative plant research. Due to a number of natural and technical reasons (e.g., inhomogeneous scene illumination, shadows, and reflections), unsupervised identification of relevant plant structures (i.e., image segmentation) represents a nontrivial task that often requires extensive human-machine interaction. Registration of multimodal plant images enables the automatized segmentation of ’difficult’ image modalities such as visible light or near-infrared images using the segmentation results of image modalities that exhibit higher contrast between plant and background regions (such as fluorescent images). Furthermore, registration of different image modalities is essential for assessment of a consistent multiparametric plant phenotype, where, for example, chlorophyll and water content as well as disease- and/or stress-related pigmentation can simultaneously be studied at a local scale. To automatically register thousands of images, efficient algorithmic solutions for the unsupervised alignment of two structurally similar but, in general, nonidentical images are required. For establishment of image correspondences, different algorithmic approaches based on different image features have been proposed. The particularity of plant image analysis consists, however, of a large variability of shapes and colors of different plants measured at different developmental stages from different views. While adult plant shoots typically have a unique structure, young shoots may have a nonspecific shape that can often be hardly distinguished from the background structures. Consequently, it is not clear a priori what image features and registration techniques are suitable for the alignment of various multimodal plant images. Furthermore, dynamically measured plants may exhibit nonuniform movements that require application of nonrigid registration techniques. Here, we investigate three common techniques for registration of visible light and fluorescence images that rely on finding correspondences between (i) feature-points, (ii) frequency domain features, and (iii) image intensity information. The performance of registration methods is validated in terms of robustness and accuracy measured by a direct comparison with manually segmented images of different plants. Our experimental results show that all three techniques are sensitive to structural image distortions and require additional preprocessing steps including structural enhancement and characteristic scale selection. To overcome the limitations of conventional approaches, we develop an iterative algorithmic scheme, which allows it to perform both rigid and slightly nonrigid registration of high-throughput plant images in a fully automated manner.

## Introduction

In the last decade, multisensory camera systems have become indispensable tools for the high-throughput screening of quantitative plant traits upon perturbation of environmental and/or molecular-genetic factors. Multimodal screening facilities enable plant scientists to generate large quantities of image data including visible light (VIS), fluorescence (FLU), near-infrared (NIR) and 3D images that are typically analyzed separately from each other. Some image modalities such as visible light or near-infrared images exhibit low contrast between plant and background image regions, which complicates automated findings of plant structures (i.e., image segmentation). Limited efficiency of existing manual and semi-automated approaches to image segmentation has been identified as the major bottleneck of quantitative plant phenotyping pipelines [1]. A combination of low- and high-contrast image modalities (e.g., fluorescence images) by means of multimodal image registration can help to overcome the limitations of unimodal image processing. Once aligned, the binary mask of a segmented FLU image can be applied for extraction of plant regions in optically more heterogeneous VIS images. Consequently, multimodal image registration is an important tool for the automatization of plant image analysis and quantitative trait derivation from high-throughput phenotyping data.

Multimodal image alignment begins with establishment of mutual correspondences between each two structurally similar but nonidentical images. Due to large variability in optical appearance of different plants as well as the same plant in different image modalities, it is not evident what kind of image features and registration algorithms can be universally applied for the alignment of different multimodal plant images.

Differences in spatial camera resolution, position and orientation can, in general, be modeled by a combination of scaling, translations, and rotations. A plethora of methods for image registration has been developed in the past, particularly in the context of biomedical image analysis [2–6]. Depending on the type of image features or intrinsic algorithmic principles, different categorizations of registration techniques have been suggested in the literature. Here, we rely on the algorithm-focused classification of registration methods into three major groups: (i) feature-point, (ii) frequency domain and (iii) intensity-based techniques.

Methods based on the matching of feature-points (FPs) are applied when corresponding image regions exhibit local structural similarity. Pairwise correspondences between two sets of feature-points are then used for calculation of geometrical transformations. Common approaches for the detection of feature-points are based on edges and corners (e.g., FAST [7], Shi and Tomasi [8], Harris operators [9], SUSAN [10]), blob detection (e.g., MSER [11], DoG, DoH), structure tensors, and generalized feature descriptions (e.g., SURF [12], HOG, SIFT [13]). The main limitation of FP methods is the difficulty in finding a sufficient number of corresponding points in similar but nonidentical images of different modaliti [14].

Another prominent approach to image alignment relies on finding correspondences in the frequency domain. For example, Fourier- or Fourier-Mellin phase correlation (PC) techniques make use of the Fourier-shift theorem, which reformulates the problem of finding a shift in Cartesian or polar system coordinates to the phase-shift of Fourier transforms [15–17]. A closer analysis of PC methods shows that they basically perform correlations of all image structures that contribute to the synchronization of Fourier phases such as edges and corners [18]. Previous works reported that PC is surprisingly robust with respect to statistical structural image noise [19–21]. This remarkable feature of PC originates from the insensitivity of inverse Fourier integrals with respect to distortions of just a few spectral bands such as high- or low-frequency noise [22]. However, PC is also known to be less accurate in the presence of multiple structurally similar patterns or considerable structural dissimilarities such as nonrigid image transformations. The necessity of additional preprocessing steps including image filtering and scaling for improved performance of multimodal image registration using PC was repeatedly reported in the previous literature [23, 24]. Downscaling to a proper size appears to improve the robustness and accuracy of image registration by suppressing modality-specific high-frequency noise, which effectively enhances image similarity [25].

Alternatively to landmarks and frequency domain features, intensity-based methods rely on maximization of global image similarity measures such as the normalized cross-correlation (NCC) [26, 27] or the mutual information (MI) [28–32]. As a dimensionless quantity, characterizing structural image similarity of the mutual information has a considerable advantage of being independent from differences between image intensity functions and histograms [33]. This property makes MI-based registration particularly suitable for image alignment that exhibits partial structural similarity but different image intensity levels.

The above registration techniques were previously applied for alignment of medical, microscopic and aerial images. Applications of image registration in the context of multimodal plant image analysis are, however, relatively scarce [34–36]. Structural differences between images of different modalities, the presence of nonuniform image motion and blurring make alignment of multimodal plant images a challenging task. Here, we investigate the performance of three registration methods by a direct comparison with manually segmented FLU and VIS plant images of different plants. The developed algorithmic scheme is, however, not limited to FLU/VIS images and can principally be applied to coregistration of other modalities (e.g., near-infrared, 3D projection images) as well. Our experimental results show limitations of conventional approaches by straightforward application to the registration of FLU/VIS plant images. Extensions of conventional algorithmic schemes are presented that allow improvement of the robustness and accuracy of image registration by application to the automated processing of large quantities of image data in the context of high-throughput plant phenotyping.

## Methods

### Image acquisition and preprocessing

**Fig. 1:**
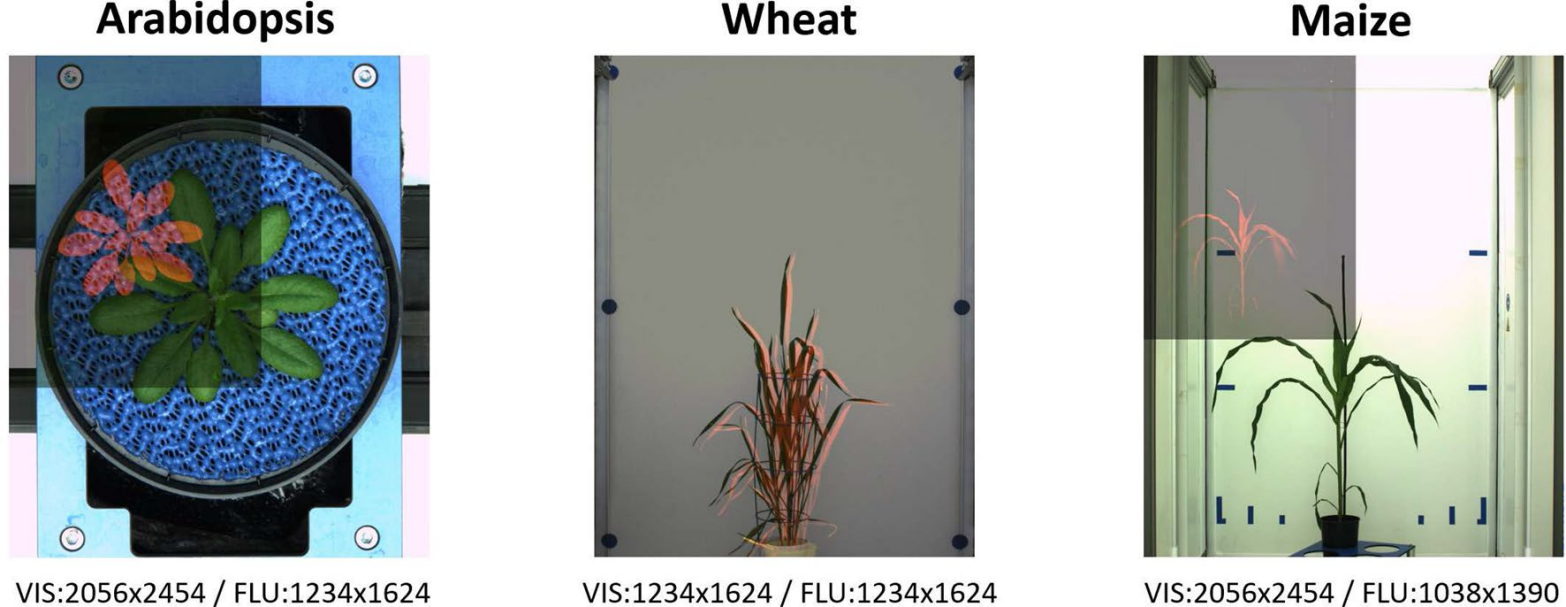
Examples of FLU/VIS images of Arabidopsis, wheat and maize shoots taken at different phenotyping facilities with different camera resolutions

**Fig. 2:**
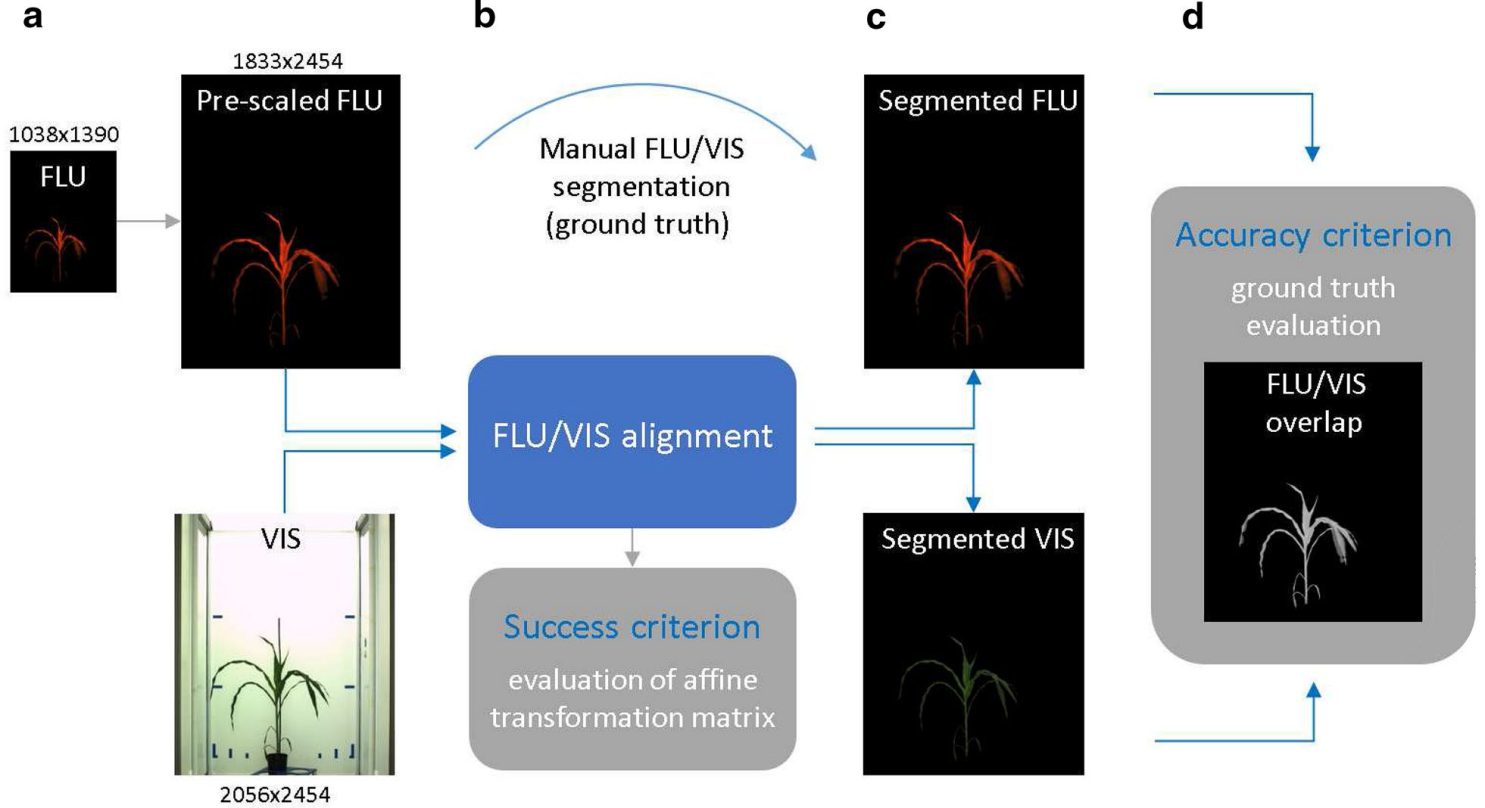
Scheme of evaluation of image registration. **a** FLU images are prescaled to the height of VIS images in order to improve robustness of subsequent registration. **b** Registration of prescaled FLU and VIS images is performed to obtain the transformation matrix $T_{ij}$ describing global image rotation, scaling and translation. Transformations ranging within the scope of admissible rotation, scaling and translation values are treated as a success; otherwise, registration is considered to have failed. **c** To assess accuracy of image registration, the resulting transformation is applied to manually segmented FLU/VIS images. **d** The accuracy of FLU/VIS image registration is measured as the overlap ratio (OR) between the area of the manually segmented VIS plant image that is covered by the registered FLU image and the total area of the manually segmented plant regions; see Eq. 2

Time-series of visible light (VIS) and fluorescence (FLU) top-/side-view mages of developing Arabidopsis, wheat and maize shoots were acquired from high-throughput measurements over more than two weeks using LemnaTec-Scanalyzer3D high-throughput phenotypic platforms (LemnaTec GmbH, Aachen, Germany). Figure 1 and Table 1 give an overview of the image data modalities and formats used in this study. To assess robustness and accuracy of image registration, investigations were performed with both original (i.e., unsegmented) and manually segmented FLU/VIS images that represent ideally filtered data free of any background structures. Manual segmentation was performed using supervised global thresholding of the background regions, followed by manual removal of any remaining structural artifacts. Since fluorescence and visible light cameras generate images of different dimensions (i.e., FLU—2D grayscale, VIS—3x2D color images), original RGB visible light images images are converted to grayscale. In addition to grayscale intensity images, registration was performed with edge-magnitude images that were calculated as suggested by [37]. Before registration was applied, FLU images were resampled to the same spatial resolution as the VIS images, which improves the robustness of image alignment algorithms, as shown in Fig. 2a. Furthermore, to study the effects of the characteristic image scale on algorithmic performance, registration was applied to both originally sized and equidistantly downscaled images, which effectively performs progressive low-pass smoothing. No further preprocessing steps were used with exception of top-view Arabidopsis images, where the contrasting blue mat was eliminated prior to image registration.

**Table 1:** An overview of image data used in this study including three different experiments of three different species, each taken in visible light and fluorescence, obtained by three different LemnaTec high-throughput phenotyping facilities for large, intermediate-size and small plants at the IPK Gatersleben

Plants/views	# plants	# days	# angles	# FLU/VIS pairs	VIS size	FLU size
Arab. T./top	4	20	1	80	2056 × 2454	1234 × 1624
Wheat/side	4	47	3	564	1234 × 1624	1234 × 1624
Maize/side	6	22	4	526	2056 × 2454	1038 × 1390

### Image registration using built-in and extended MATLAB functions

Image registration was performed using the following three groups of registration routines, as provided with the MATLAB 2018a Image Analysis toolbox (The MathWorks, Inc., Natick, Massachusetts, United States):

- For feature-point matching, several different edge-, corner- and blob-detectors were used. In addition to built-in MATLAB functions that rely on one particular feature detector, an integrative multifeature generator was introduced that merges the results of different feature-point detectors.
- Alternative image registration techniques based on frequency domain features rely on the MATLAB *imregcorr* function, which performs Fourier-Mellin phase correlation of the corresponding spectral image transforms. For assessment of image transformation reliability, a fixed threshold of the maximum PC peak height (i.e., $H>0.03$) was used as suggested in [16]. Transformations obtained with $H<0.03$ typically indicate a failure of PC registration, for example, due to excessively low and missing structural similarities between two images.
- The third method of image registration is based on maximization of the Mattes mutual information between each two images using the MATLAB *imregister* function [30, 31].

All registration methods were applied to determine a global rigid transformation including rotation, scaling and translation, which correspond to the ‘similarity’ option of MATLAB transformation routines; see an overview in Table 2.

**Table 2:** Overview of three groups of image alignment methods including feature-point (FP) matching, phase correlation (PC) and image intensity (mutual) information (INT) image features and corresponding MATLAB functions used for calculation of pairwise image correspondences

Method	Feature	MATLAB function	References
PC	Phase correlation	imregcorr	[15–17]
INT	Mutual information	imregtform	[28–32]
FP:BRISK	Corners	detectBRISKFeatures	[38]
FP:FAST	Corners	detectFASTFeatures	[7]
FP:Harris	Corners	detectHarrisFeatures	[9]
FP:KAZE	Blobs	detectKAZEFeatures	[39]
FP:MinEigen	Corners	detectMinEigenFeatures	[8]
FP:MSER	Intensities	detectMSERFeatures	[11]
FP:SURF	Blobs	detectSURFFeatures	[12]

All methods are used with the ‘similarity’ option, which restricts the class of possible image transformations to a combination of global rotation, scaling and translation

### Evaluation of image registration

To evaluate the results of image registration, two criteria for characterizing the robustness and accuracy of image alignment are used.

#### Success rate of image registration

To assess the robustness of image registration, the success rate (SR) is calculated as the ratio between the number of successfully performed image registrations ($n_s$) and the total number of registered image pairs (*n*):

1 $$\begin{aligned} SR = \frac{n_s}{n}. \end{aligned}$$

Image registration was defined as successful when components of the transformation matrix lay within a range of admissible values of translation ($|T|<300$ pixels), rotation ($|\cos (\alpha )|<0.15$) and scaling ($S\in [0.75,1.25]$). Geometrical transformations that do not fit in this range were treated as a failure of image registration.

#### Accuracy of image registration

The second criterion is constructed to quantify the accuracy of image registration. For this purpose, geometrical transformations acquired for a pair of FLU/VIS images are applied to manually segmented images, and the overlap ratio (OR) between the area of VIS plant regions covered by the registered FLU image ($a_r$) and the total area of manually segmented plant regions (*a*) in VIS image is calculated, as shown in the scheme of evaluation of image registration in Fig. 2:

2 $$\begin{aligned} OR = \frac{a_r}{a}. \end{aligned}$$

Asymmetric definition of OR, which considers only VIS images, was used because the primary goal of FLU/VIS registration consists of segmentation of plant regions in VIS images.

## Experimental results

First, the built-in MATLAB routines for feature-point (FP)-, phase correlation (PC)- and intensity (INT)-based image registration were applied for alignment of original (i.e., unscaled, unfiltered) FLU and VIS images of developing Arabidopsis, wheat and maize shoots. The results of this first feasibility test show a superior success rate of INT registration in comparison to FP- and PC-based approaches; see Table 3. However, the accuracy of INT registration exhibits substantial variations among different plant species.

**Table 3:** Success rates and accuracy ratios of the successful alignment of originally sized Arabidopsis, wheat, and maize FLU/VIS images using FP/PC/INT registration techniques

	Success rate (%)			Accuracy (%)		
	FP	PC	INT	FP	PC	INT
Arabidopsis	44.44	11.11	96.30	43.50	49.29	93.27
Wheat	81.77	13.45	97.52	66.39	67.28	85.40
Maize	89.15	97.79	86.58	70.12	77.99	63.57

**Fig. 3:**
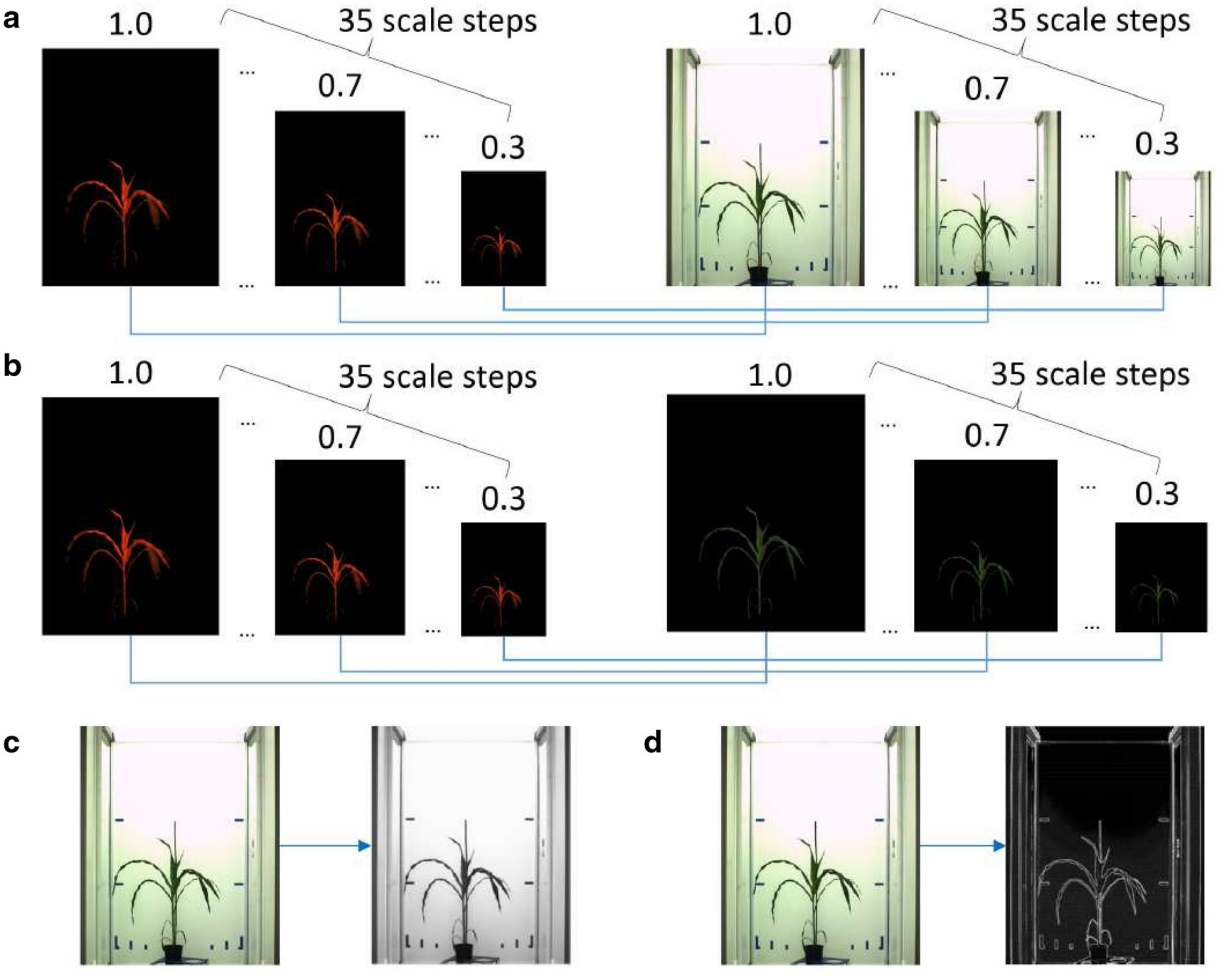
Overview of preprocessing conditions (filtering and scaling) for evaluation of plant image registration using FP, PC and INT methods. 35 scale steps in the range between [0.3, 1.0] (step size = 0.02) were probed with original (**a**) and manually segmented (**b**) FLU/VIS images. Since registration routines require grayscale images, RGB images are converted to grayscale (**c**). In addition, registration is performed with color-edge FLU/VIS images (**d**)

**Fig. 4:**
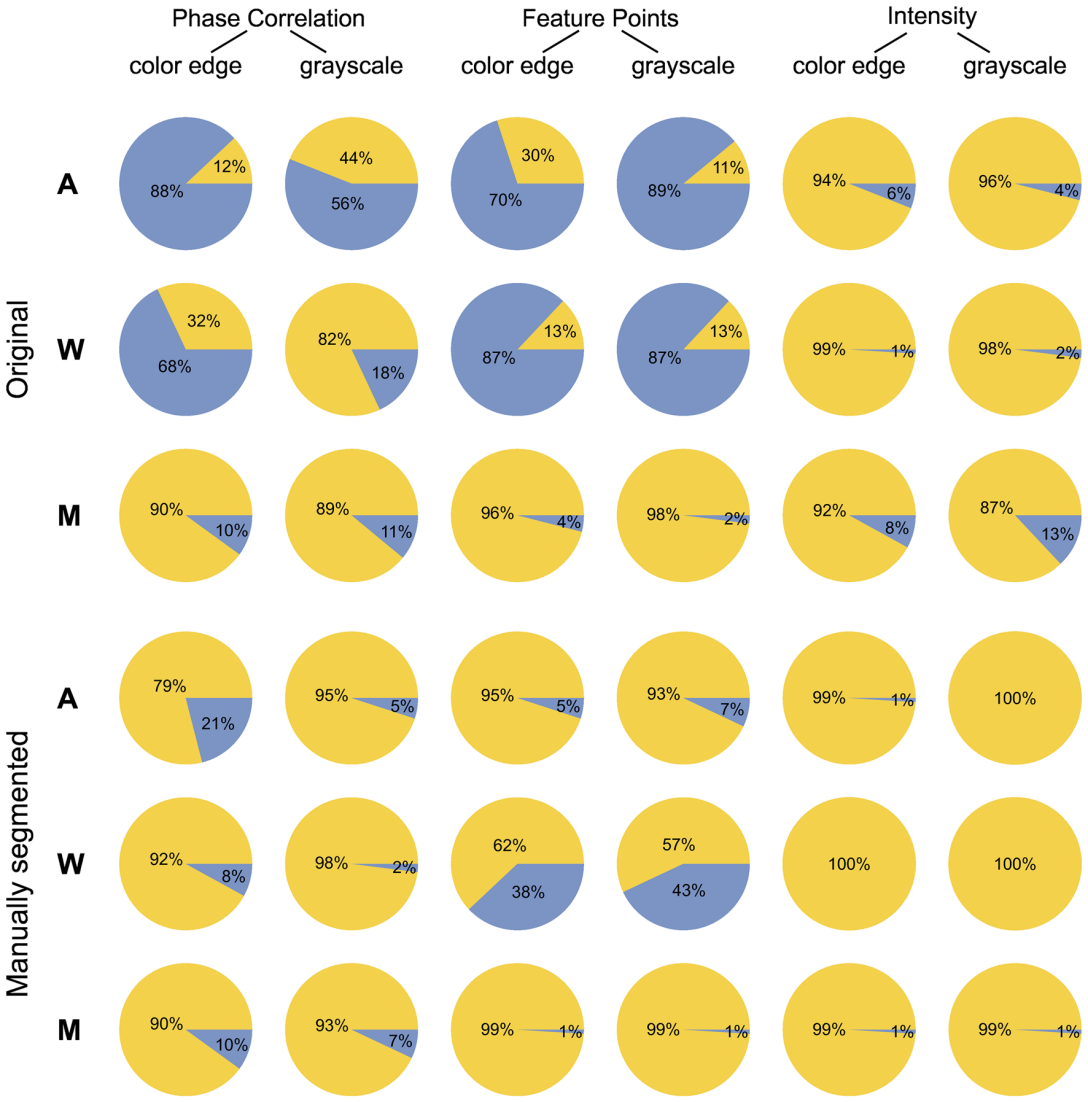
Summary of effects of background filtering on the success rate of FP, PC and INT registration of FLU/VIS images of Arabidopsis, wheat and maize plant shoots. Pie charts show the percentage of successful (yellow-colored fractions) and failed (blue-colored fraction) registration of original and manually segmented grayscale and color-edge FLU/VIS images

To dissect possible causes of reduced robustness and accuracy of image registration methods by application to original FLU/VIS images, a systematic analysis of the effects of structural image enhancement and scaling was performed. Figure 3 gives an overview of the preprocessing conditions that were evaluated with respect to image registration outcome, including 35 equidistant downscaling steps in the range of scaling factors [0.3, 1.0], as well as grayscale (GS) and color-edge (CE) representations of original and manually segmented FLU/VIS images. Figure 4 summarizes statistics of success rates (SRs) of FP, PC, and INT registration by application to original (i.e., unscaled, unfiltered) and manually segmented (ground-truth) plant images. From this overview, it is evident that removal of background structures significantly improves the robustness of image registration, i.e., the number of image registrations with admissible transformations.

**Fig. 5:**
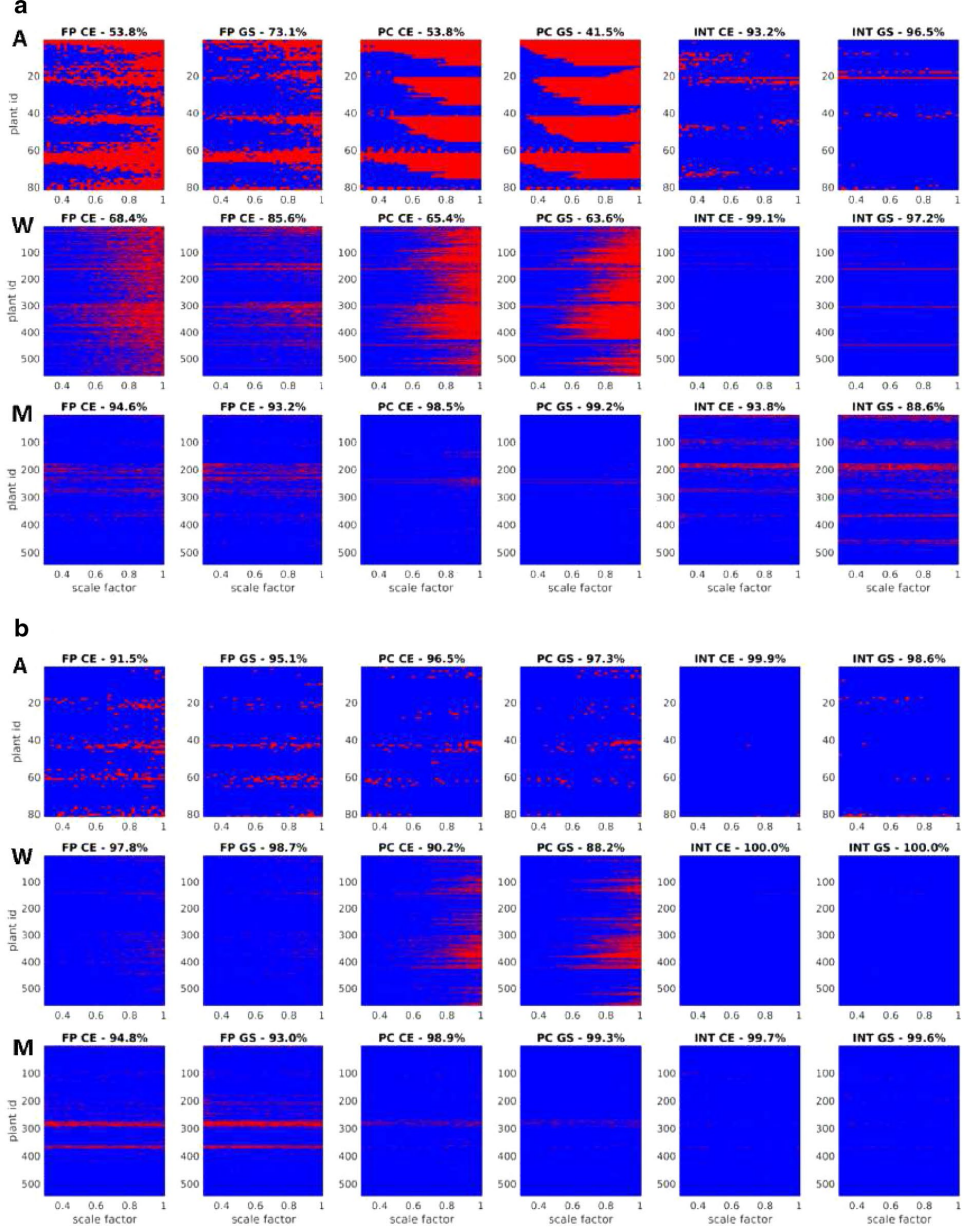
Summary of effects of image downscaling on the success of FP, PC and INT registration of FLU/VIS images of Arabidopsis (A), wheat (W) and maize (M) plant shoots. Color diagrams show formally successful registrations (blue) and obvious misalignments (red) of original (**a**) and manually segmented (**b**) images with dependency on the downscaling ratio ranging between [0.3, 1.0]. Diagram titles indicate registration techniques (FP, PC, INT), type of image modality (color-edges—CE, grayscale—GS) and the overall success rate of image registration for a particular method, image modality and plant species

**Fig. 6:**
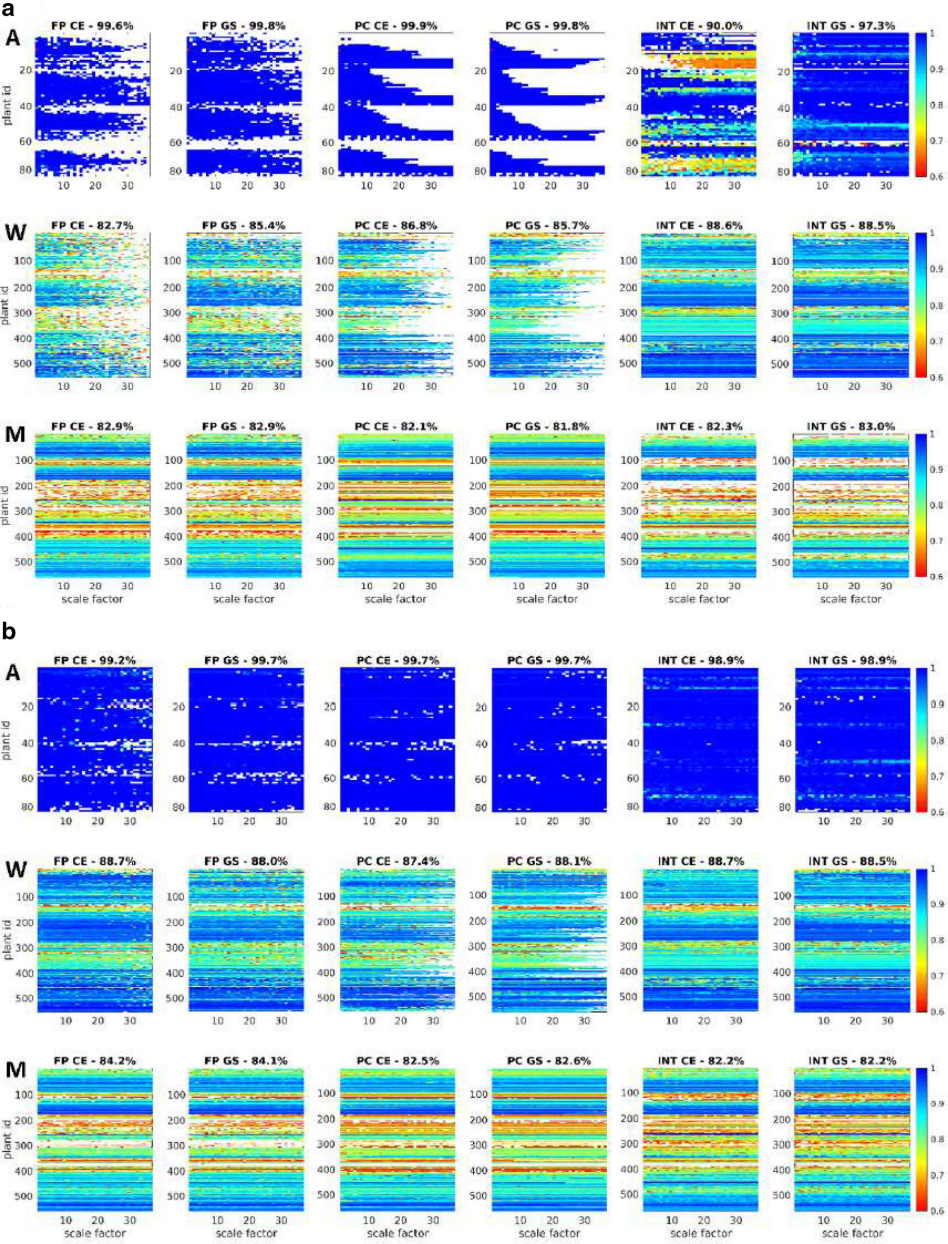
Summary of effects of image downscaling on the accuracy of FP, PC and INT registration of FLU/VIS images of Arabidopsis (A), wheat (W) and maize (M) plant shoots in comparison to manually segmented (ground-truth) data. Color diagrams show the accuracy ratio (Eq. 2) of registration with dependency on image downscaling. White areas in the color diagrams correspond to FLU/VIS misalignments, i.e., red areas in Fig. 5

To dissect the effects of characteristic image scale on the results of image registration, equidistant downscaling of FLU/VIS images in the range of scaling factors between [0.3, 1.0] was applied. Figures 5 and 6 show a summary of success rate and overlap ratio calculations for time-series of developing Arabidopsis, wheat and maize shoots. As seen in the FP/PC diagrams of Fig. 5a, the FP and PC methods exhibit reduced success rates of registration for originally sized and moderately downscaled images. Background filtering in manually segmented images significantly improves the success rate of FP and PC registration; see Fig. 5b. Among these techniques, INT registration shows the most robust performance in terms of SR.

Complementary plots of registration accuracy in Fig. 6 measured using Eq. 2 indicate, however, that a formally successful image alignment within the range of admissible transformations is not always associated with a good overlap between registered and manually segmented (ground-truth) plant areas. In particular, exceptionally high SR values of INT-based registration (Fig. 5) are not accompanied by high OR. Further, one can see that some plant images (e.g., Arabidopsis, top view) can be generally aligned more accurately than the others (e.g., wheat, maize, side view). Thereby, the deviation of registered plant areas from the ground-truth data is larger for original images in comparison to manually segmented plants, cf. Fig. 6a versus b.

**Fig. 7:**
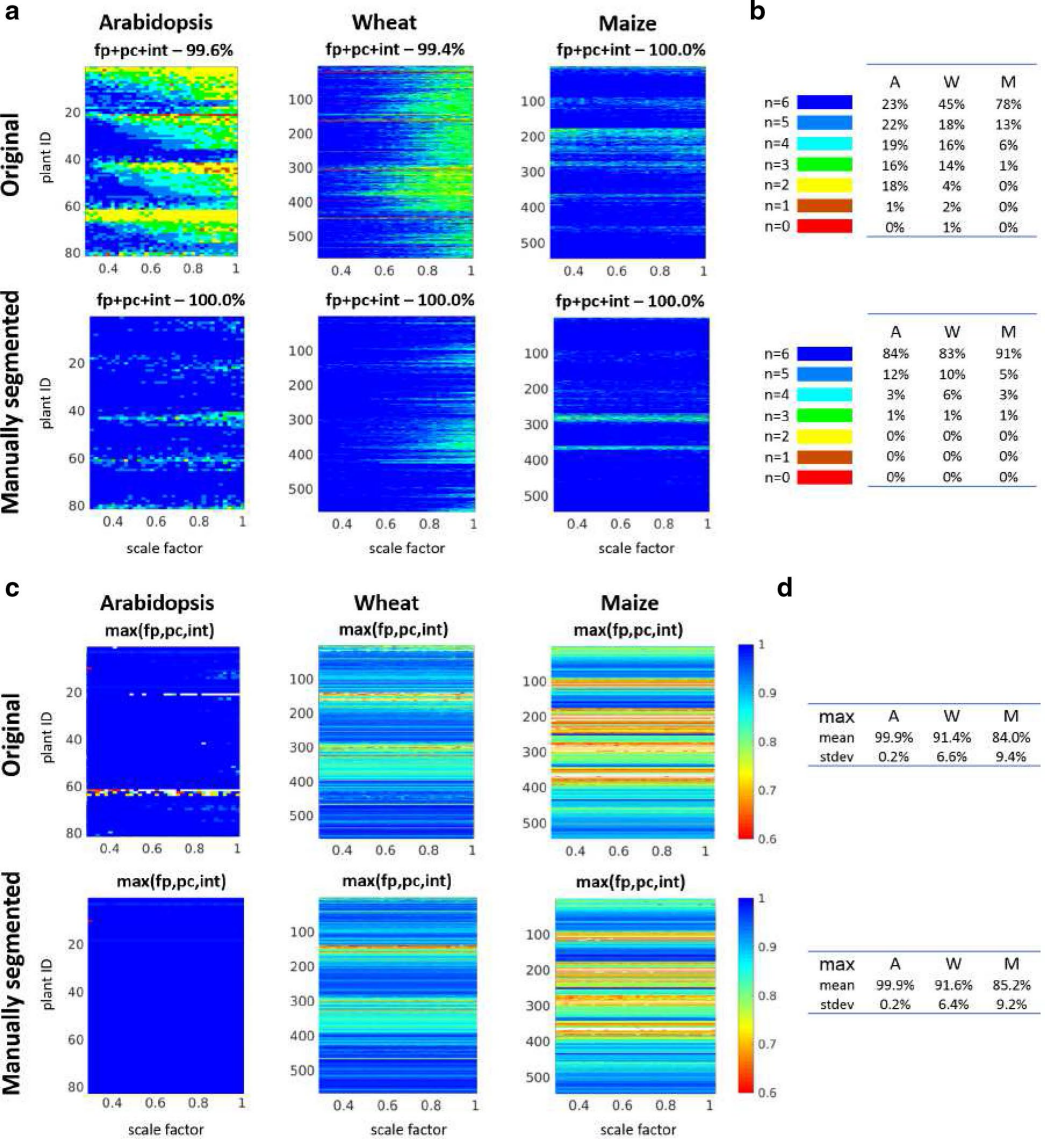
Statistics of success and accuracy of FLU/VIS registration by combined application of different methods and image representations. **a** Color mapping of the number methods with successful image registration. **b** Color codes indicate the number of methods successfully performing FLU/VIS registration with dependency on the scaling factor. Diagram titles show the percentage of successfully registered images by combination of all methods (FP, PC, INT) and image representations (color-edges—CE, grayscale—GS). **c** Color mapping of maximum accuracy among FP-CE, FP-GS, PC-CE, PC-GS, INT-CE, INT-GS. **d** Mean/Stdev values of accuracy for plant species

Figure 7 shows success and accuracy statistics of image registration by combined application of all three methods (FP, PC, and INT) and both image representations, i.e., grayscale (GS) and color-edge (CE) images. From this diagram, it is evident that the majority of FLU/VIS image pairs can be successfully registered with more than one method and preprocessing condition. However, there are also some cases where only a few or even only one particular method is capable of successfully performing FLU/VIS image alignment. Again, background filtering in manually segmented images significantly improves success rates by combined application of different registration techniques; see Fig. 7a, b. To quantify the advantage of combined image registration, the maximum accuracy among all six techniques (i.e., FP-CE, FP-GS, PC-CE, PC-GS, INT-CE, and INT-GS) is calculated. From Fig. 7c, it is clearly visible that some plants (e.g., Arabidopsis) can generally be registered more accurately by one single registration step than others, and background elimination decisively improves the accuracy performance of FLU/VIS registration.

**Fig. 8:**
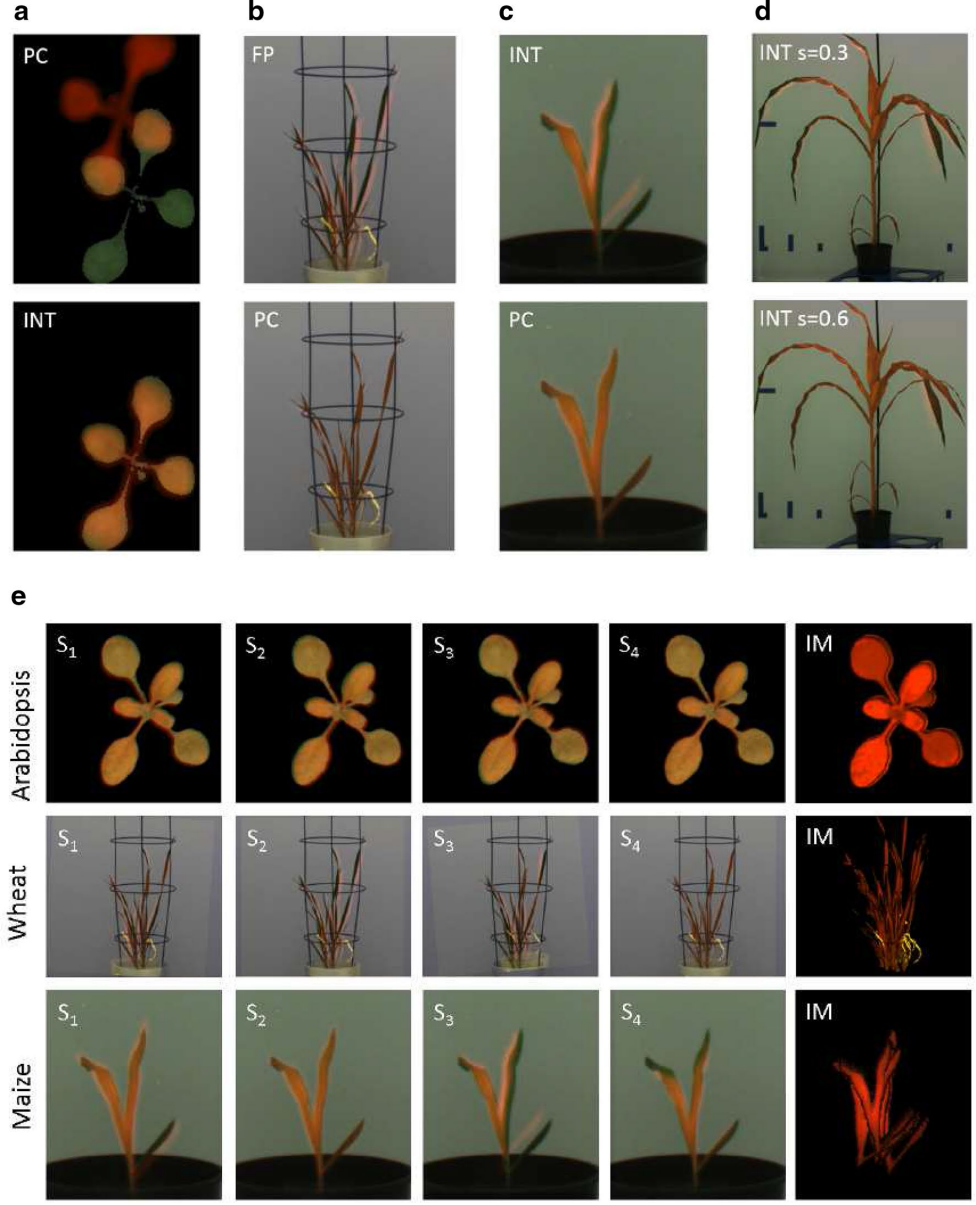
Examples of FLU/VIS image misalignments due to **a** multiple similar leaves, **b**, **c** nonuniform leaf motion, **d** blurring in FLU channel, and optimal registration of the same images using other methods or image preprocessing conditions. **e** Single-step registrations of differently scaled images (S$_i$) may result in partial alignment of plant structures. To improve the accuracy of registration for nonuniformly moving plant structures, the results of multiple registrations are integrated into a single integrated mask (IM)

A closer analysis of cases with low OR revealed several possible causes for inaccurate FLU/VIS alignment including repeated patterns (e.g., multiple similar leaves) and nonuniform image motion due to inertial movements of leaves. Different registration methods exhibit different tolerance levels with respect to structural image distortions. For example, PC registration turns out to be particularly sensitive to multiple self-similar patterns such as leaves of similar shape and size; see Fig. 8a. Finding complementary feature-points in FLU/VIS images appears to be particularly difficult for thin moving leaves of wheat shoots; see Fig. 8b. Intensity-based registration can, in turn, be misled by the intensity of background structures similar to intensity of shoots; see Fig. 8c. Finally, one and the same method may produce alignments of different accuracy with differently scaled and preprocessed images; see Fig. 8d.

**Fig. 9:**
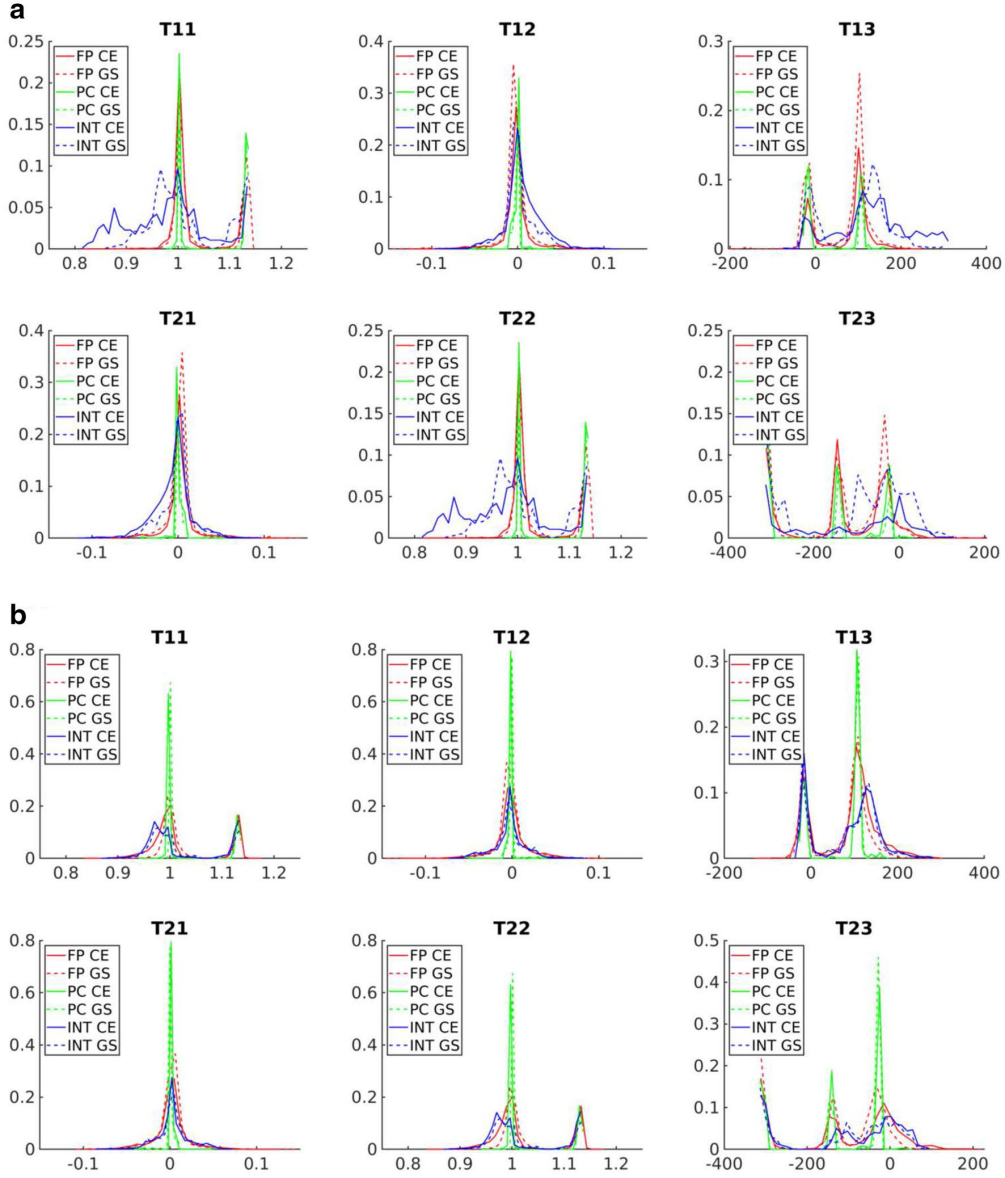
Distributions of components of the transformation matrix ($T_{ij}$) acquired by registration of FLU/VIS images of Arabidopsis shoots using different methods (FP, PC, INT), different image representations (color-edges—CE, grayscale—GS) and different scaling factors. The $2\times 2$ submatrix of $i,j<3$ values describes scaling and rotational components of $T_{ij}$, while $T_{13}$ and $T_{23}$ are the global image translations

Depending on image preprocessing, registration algorithms may calculate quite different image transformations. Figure 9 shows component distributions of the transformation matrix that were assessed with different registration techniques and preprocessing conditions (i.e., scaling factors, background filtering). As one can see, the values of scaling, rotation and translation undergo considerable variations that correspond to both optimal and suboptimal FLU/VIS image alignments, such as those shown in Fig. 8. At first glance, registration dependency on structural image content and preprocessing appears to be disadvantageous. However, it turns out to be a very helpful feature. Here, we exploit the variability of geometrical transformations resulting from optimal and suboptimal image registration to construct an integrated registration mask that allows for a piecewise approximation of nonuniformly moving plant regions that otherwise could not be completely covered by a single-step registration; see Fig. 8e.

**Fig. 10:**
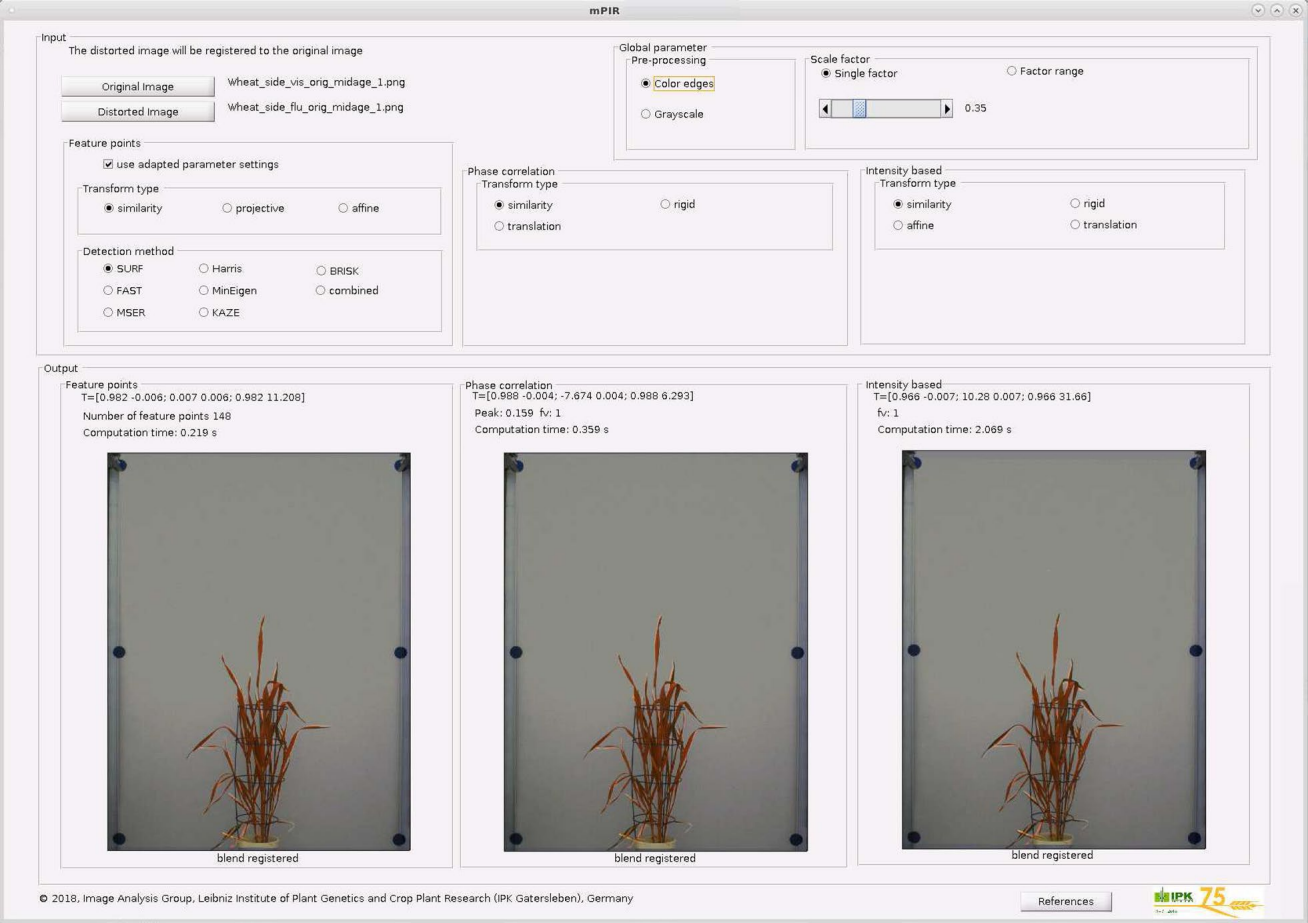
Overview of the GUI interface of the multimodal plant image registration tool (mPIR) for demonstration of FLU/VIS image alignment using FP/PC/INT registration algorithms with different parameter settings

Computational costs of pairwise image registration are essentially dependent on image size, type of registration method and diverse algorithmic parameters. To demonstrate the above-described parameter-dependent performance of FP/PC/INT registration techniques for the automated alignment of multimodal plant images, a GUI software tool with examples of plant images is provided for direct download from our homepage;^1^ a screen shot is shown in Fig. 10. While the performance of image registration algorithms was primarily evaluated with FLU and VIS images, our exemplary tests show that they are also applicable to fusion of other image modalities, e.g., FLU/NIR or VIS/NIR. Examples of FLU, VIS and NIR plant images are included in our online file repository.

## Conclusion

Multimodal image registration opens new possibilities for the automatization of image segmentation and analysis in high-throughput plant phenotyping. Using image registration, the result of a straightforward FLU image segmentation can, for example, be applied to automatically detect plant regions in optically more heterogeneous visible light images. Furthermore, the spatial alignment of different image modalities paves the way for consistent assessment of a multiparametric plant phenotype including information on local chlorophyll/water content and disease-/stress-related pigmentation. Our experimental results using three common registration techniques (FP, PC, and INT) show that the robustness and accuracy of FLU/VIS image alignment undergo substantial variations depending on the plant species, interplay between the background and plant intensities, and image preprocessing conditions. In general, background filtering, structural enhancement and downscaling significantly improve the performance of FLU/VIS image registration. However, none of the methods and preprocessing conditions offers universal advantages that guarantee optimal results of single-step registration by application to arbitrary image data. On the basis of insights gained in this study, we conclude that a combination of different registration techniques, scaling levels and image representations (i.e., grayscale and color-edge) enables significantly more robust and accurate results to be obtained when compared to single-step image alignment using one particular method and/or one particular image preprocessing filter. We began this study with the assumption of global rigid image transformations. However, it turned out that FLU/VIS images may exhibit nonuniform motion due to uncorrelated inertial movements of tillers and leaves after relocation or rotation of plant carriers during stepwise image acquisition. Integration of multiple registration results obtained for different preprocessing conditions into one single integrated mask allows this problem to be overcome by constructing a piecewise approximation of nonuniform image motion, which otherwise would require the application of significantly more expensive nonrigid registration.

The basic approach to automated alignment of plant images using a combination of feature detectors and preprocessing conditions presented in this work was evaluated with fluorescence and visible light images, but the results can principally be applied to coregistration of other image modalities, e.g., near-infrared images.

## Footnotes

1 https://ag-ba.ipk-gatersleben.de/mpir.html.
